# Next-Generation Healthcare: Artificial Intelligence Applications in Disease Management

**DOI:** 10.3390/diagnostics14111087

**Published:** 2024-05-24

**Authors:** Sami Akbulut, Cemil Colak

**Affiliations:** 1 Department of Surgery and Liver Transplant Institute, Inonu University Faculty of Medicine, Malatya 244280, Turkey; 2 Department of Biostatistics, Bioinformatics and Medical Informatics, Inonu University Faculty of Medicine, Malatya 44280, Turkey; cemil.colak@inonu.edu.tr

## 1. Introduction

The quick and large development in the accumulation of medical data provides broad potential for the application of artificial intelligence technologies. This Special Issue highlights the cutting-edge artificial intelligence innovations that are revolutionizing the healthcare business. Through the usage of artificial intelligence, conventional healthcare systems are becoming mechanized, resulting in better operational efficiency. Furthermore, artificial intelligence plays a significant purpose in accelerating diagnostic procedures, permitting faster and more precise diagnoses of medical diseases. Moreover, artificial intelligence plays a key role in optimizing treatment regimens, leading to better health outcomes for patients. This Special Issue presents a full assessment of these innovative advances, illustrating the increasing importance of artificial intelligence as a critical tool in current healthcare. The exponential increase in medical data is transforming healthcare through artificial intelligence technologies, boosting operational efficiency and patient outcomes. Artificial intelligence applications in robotics, computer vision, and natural language processing are transforming healthcare service and research [1,2]. These improvements enable quicker and more precise tests, individualized treatment strategies, and they allow us to make efficient discoveries in medicine. Artificial intelligence’s impact extends to optimizing hospital operations, easing administrative duties, and improving resource allocation, eventually enhancing patient experiences, and reducing costs [3,4]. The availability of huge data supported by limitless cloud storage promotes the creation of artificial intelligence applications, training algorithms to interact with practice data for enhanced diagnosis and therapy outcomes. As artificial intelligence continues to evolve, it is necessary to address concerns such as data privacy, algorithmic biases, and ethical considerations to ensure its safe and effective incorporation into healthcare systems [3,4,5].

## 2. Brief Overview and List of Contributions

- Blood Cancers: The work on this topic showcases a revolutionary deep-learning approach for accurately diagnosing acute lymphoblastic leukemia (ALL) in blood pictures. This approach transcends standard methods by employing feature selection algorithms to extract the most informative data from complicated pictures.
- Infectious disorders: Another study introduces a framework that leverages machine learning to identify Heparin-binding protein (HBP), a biomarker critical for diagnosing infectious disorders. This technique showed great accuracy in differentiating HBP from non-HBP samples.
- Diabetic Macular Edema (DME): The study on this analyzes how artificial intelligence can aid in predicting DME, a consequence of diabetes that can lead to eyesight loss. The suggested method employs knowledge graphs to adjust for missing data and strengthen disease prediction models, promoting early intervention.
- Oral Squamous Cell Carcinoma (OSCC): Researchers have created a nomogram prediction model utilizing artificial intelligence to assess the prognosis of OSCC patients after surgery. This model integrates many criteria to predict survival rates, aiding in individualized treatment regimens.
- Drug-induced Hepatotoxicity: The ability of artificial intelligence to examine big datasets is applied to uncover potential biomarkers for detecting hepatotoxicity, a liver ailment caused by certain medicines. This paves the path for the creation of more efficient diagnostic instruments.
- Mandibular Length Prediction: Artificial intelligence algorithms are proven to be adept at predicting face growth patterns. This work reveals how machine learning can effectively estimate post-pubertal mandibular length, a crucial tool in orthodontics.
- Appendicitis Diagnosis: Another use of artificial intelligence tackles the difficulty of diagnosing appendicitis. A machine learning approach is presented to identify between perforated and non-perforated appendicitis with excellent accuracy, aiding in key therapeutic choices.
- Diabetes Detection: The issue discusses a revolutionary deep learning strategy for early diabetes detection. By turning numerical data into visual graphs, the study reveals the efficiency of artificial intelligence in recognizing the disease at an earlier stage.
- Brain Tumor Detection: The fight against brain cancers is reinforced by artificial intelligence-powered analysis of Magnetic Resonance Imaging (MRI) data. This study compares multiple machine learning models to discover the most effective technique for early and accurate brain tumor diagnosis.
- Inflammatory Bowel Disease (IBD): The potential of artificial intelligence in addressing IBD is investigated. The issue highlights the prospects of artificial intelligence in interpreting genomic data, predicting disease progression, and assessing therapy response, ultimately enhancing patient care.
- Breast Cancer Diagnosis: A systematic study of artificial intelligence applications in breast cancer diagnosis is presented. The paper stresses the rising amount of research in this field, with Convolutional Neural Networks (CNNs) emerging as a preferred artificial intelligence model for breast cancer screening.

## 3. Beyond Disease Detection: Artificial Intelligence’s Expanding Role in Medicine

The uses of artificial intelligence in medicine extend far beyond disease detection. Artificial intelligence is being investigated for jobs, such as

- Drug Discovery: artificial intelligence can analyze vast datasets of molecular structures to identify potential drug candidates, accelerating the drug development process.
- Robotic Surgery: artificial intelligence-powered robotic surgery systems can improve precision, minimize invasiveness, and enhance patient outcomes.
- Personalized Medicine: artificial intelligence can analyze a patient’s individual medical data to recommend the most effective treatment options.
- Medical Imaging Analysis: artificial intelligence algorithms can analyze medical images, such as X-rays and CT scans, to detect abnormalities and aid in diagnosis.
- Epidemic Prediction and Prevention: artificial intelligence can be used to analyze vast amounts of data to identify patterns and predict disease outbreaks, enabling preventative measures [3].

## 4. Challenges and Ethical Considerations

Even though artificial intelligence has a lot of potential for the future of medicine, there are obstacles to overcome. These include the following:

- Data Privacy and Security: ensuring the privacy and security of sensitive patient data is critical.
- Algorithmic Bias: Artificial intelligence models can perpetuate biases present in the data they are trained on. Mitigating bias is essential for ensuring fair and equitable healthcare.
- Transparency and Explainability: it is crucial to understand how artificial intelligence models arrive at their decisions, particularly in high-stake medical scenarios.
- Regulatory Frameworks: regulatory frameworks need to be developed to ensure the safe and ethical use of artificial intelligence in medicine [4,5].

## 5. Conclusions

The potential of artificial intelligence to revolutionize healthcare is genuinely transformational. As artificial intelligence technology continues to advance and we overcome our present challenges, we should expect big breakthroughs in various domains of medicine. This involves earlier illness prevention, more accurate diagnosis, and the development of individualized treatment approaches. Ultimately, this will lead to improved patient outcomes and a healthier population. The future of medicine promises tremendous cooperation between human knowledge and artificial intelligence. Physicians will make use of artificial intelligence’s capabilities to examine vast volumes of patient data, identify intricate patterns, and gain a greater understanding of patient issues. This will open the door to a new era of personalized medicine, in which a patient’s treatment plan will be tailored to their unique needs and genetic composition. Using artificial intelligence as a powerful tool, medical professionals may diagnose patients more accurately, spot potential health risks, and recommend the best course of action for each patient [6].
